# Health Benefits Derived from Forest: A Review

**DOI:** 10.3390/ijerph17176125

**Published:** 2020-08-23

**Authors:** Gianluca Grilli, Sandro Sacchelli

**Affiliations:** 1 Department of Agriculture, Food, Environment and Forestry, University of Florence, I-50144 Florence, Italy; Gianluca.Grilli@esri.ie; 2 Economic and Social Research Institute, D02 K138 Dublin, Ireland; 3 Trinity College Dublin, D02 PN40 Dublin, Ireland

**Keywords:** forest therapy, forest recreation, relaxation, stress relief, quantitative analysis, psycho-physiological indicators

## Abstract

In this paper the scientific literature on the association between forests, stress relief and relaxation is reviewed with the purpose to understand common patterns of research, the main techniques used for analysis, findings relevant to forest-therapy-oriented management, and knowledge gaps. The database of studies was collected with a keyword search on the Web, which returned a set of 32 studies that were included in the analysis. The main findings and patterns were identified with a text mining analysis of the abstract to search for keyword patterns across studies. The analysis indicates that most studies compared rest and relaxation performances across urban and forest environments and used a combination of self-reported measure of stress or rest collected with validate scales, e.g., the Profile of Mood of States (POMS) and the Restoration Outcome Scale (ROS), and a minority-only set of these two groups of indicators. Results of this review indicate that primary studies identified a positive association between forest exposure and mental well-being, in particular when compared to urban environments, thus suggesting that forest are effective in lowering stress levels. This study found that, to date, the characteristics of forests and characteristics of the visit are little investigated in the literature. For this reason, more research with a focus on forest variables such as tree species composition, tree density and other variables affecting forest landscape should be further investigated to inform forest management. Similarly, the characteristics of the visits (e.g., length of visit and frequency) should be further explored to provide robust forest therapy guidelines.

## 1. Introduction

Recreation is a very important ecosystem service provided by natural areas. In particular, forested areas provide countless recreational opportunities such as hiking, picnicking, mushroom and berry picking, biking and horse riding. For this reason, there is an increasing trend in forest management to leave some forested areas as dedicated areas for recreation. The propensity of a forest for recreation is highly specific [1]. Previous research highlighted the importance of forest structure for recreation; in particular, it has been observed that tree species composition, forest cover and forest structure are important variables [2,3]. With respect to forest types, mixed and coniferous forests are often preferred destinations for recreational purposes compared to broadleaf forests [4,5], while canopy cover has been found to influence recreation based on transport and access to forests, with lower canopy cover preferred by motorized recreationalists and higher density cover by non-motorized visitors [6]. The other examined preference studies linked forest structure in terms of shrubs, dead wood, and type of higher trees to different preferences in terms of recreational activities [7].

One of the recent trends in the recreational aspects of forest management is to explore the potential of woodlands for stress relief and rest. This is especially important for forested areas in proximity of cities. Urban areas are increasingly seen as a source of stress, because industrialized society has created a frenetic routine for individuals. Urbanization is increasing, particularly in some developing nations, and, as a result, it is estimated that 67% of the world population will be living in urban areas by 2050 [8]. Easy access to oases where one can rest and that are close to urban areas is important for resting the mind and mental well-being. The health benefits of being in contact with nature are demonstrated by several studies, many of which were collected in the meta-analysis published by Gascon et al. [9]. More recently, a study in England found that spending 120 min per week in nature is sufficient to maintain good health and well-being levels [10]. The benefits of green areas for well-being are not necessarily linked to active visitation, as the simple exposure to natural environments is equally beneficial. In fact, several hospitals are now equipped with the so-called “healing gardens”, where patients can spend some time to recover from mental diseases [11,12,13]. Due to the close link of stress to urban life, most studies analyse the impact of within-city green spaces on mental well-being. Bowler et al. [14] collected all studies specifically designed to understand the effect of urban green spaces on rest in a systematic meta-analysis and found that all primary sources identified some degree of effectiveness of green spaces on human health. While urban green spaces are recognized environments where one can rest, the potential of close-to-city forests for therapy is less explored, and little effort has been dedicated to gather the existing scientific evidence to identify lessons learned and knowledge gaps.

With this in mind, this contribution offers a review of the literature exploring the association between forest exposure and mental well-being, in particular with respect to relaxation or rest from stress. The main objective of the paper is to provide a snapshot of the existing knowledge, techniques and approaches implemented to study forest therapy. The analysis considers stress indicators, forest stands, and forest characteristics associated with relaxation, as well as interesting avenues for future research. Results are useful to indicate development paths for forest managers interested in forest therapy and to inform researchers and analysis on evidence that is still missing on this relevant topic.

## 2. Materials and Methods

### 2.1. Database Collection

**Figure 1:**
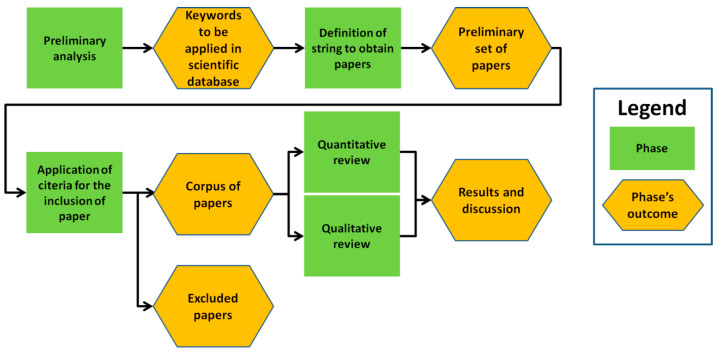
General methodological framework of the study.

The data collection was carried out with a keyword search in Scopus, i.e., one of the largest scientific databases available. Scopus was chosen because it is user-friendly and large enough to include most papers indexed in other famous servers, for example Web of Science. A preliminary qualitative study of four papers relevant to the topic [15,16,17,18] was undertaken to familiarize with the specific nomenclature and select the most appropriate keywords. The keyword search was then conducted using one word among “forest”, “woodlands” or “greenwoods” in combination with one of the following: “therapy”, “stress”, “relaxation”, “restoration”, “bathing” (the Scopus search was conducted on 7th August 2020 with the following string: (TITLE-ABS-KEY (forest) OR TITLE-ABS-KEY (woodlands) OR TITLE-ABS-KEY (greenwoods) AND TITLE-ABS-KEY (therapy) AND TITLE-ABS-KEY (stress) OR TITLE-ABS-KEY (restoration) OR TITLE-ABS-KEY (relaxation) OR TITLE-ABS-KEY (bathing)). The criteria for the inclusion of a contribution were the following: (1) the paper must be focused on benefits obtained from exposure to forests; (2) the paper must explore the effects of relaxation or rest of forests; (3) the paper must follow a treatment–control experiment approach; (4) the paper must use self-rated or physiological indicators of stress/rest. These criteria were used to obtain a homogeneous set of manuscripts, which were comparable in terms of topic and methodological approach. Manuscripts that did not satisfy all of these selection criteria were excluded from the analysis. For example, many contributions concentrate on urban green spaces because they are important for rest from city life. A large list of these papers has been collected previously [14]. Since one of the main objectives of this review is to provide indications for therapy-oriented forest management, urban green space papers were excluded due to the limited usefulness for forest management purposes. Finally, both quantitative (based on text mining approach) and qualitative reviews (focused on the applied methods, the effects of relaxation and rest of forests as well as influence of forest characteristics on stress recovery) were performed (Figure 1).

### 2.2. Text Mining Analysis

A quantitative evaluation was performed to integrate the qualitative literature analysis, which should be considered as a preliminary test to be integrated in future evaluations. This quantitative analysis is a text mining exercise based on the title, abstract and keyword of all examined papers (corpus). The corpus was imported as a .txt file and text mining was performed by means of the software T-Lab (www.tlab.it), a tool based on the lexicometric approach [19]. Based on the big data framework, text mining derives patterns within the corpus such as in clustering, concept extraction and sentiment analysis. In our work, we used the multidimensional scaling approach (MDS) [20]. In MDS, through the application of Sammon’s algorithm [20], relationships among lemmas in the corpus are represented in a graphical way (distance and position). Lemmas are denoted by circles of single or aggregated words. The size of the circle highlights the weights of the lemma in the corpus. The significance of the MDS map is revealed by the stress index that measures the difference between the observed dissimilarity matrix among the lemmas and the estimated one.

## 3. Results and Discussion

The search provided an initial list of 162 documents, but many papers did not satisfy all the considered criteria. After reading the abstracts, the list of documents was further cleared, and the final set included 36 papers, which were homogeneously organized and adequate for the purpose of this review. Most self-reported scales used in primary studies are known in the scientific literature with acronyms and abbreviations, which are reported in Table 1. The full list of study is shown in Table 2. Out of these documents, 24 were carried out considering measures regarding self-rated stress or rest, 27 focused on physiological measures, and 17 used both approaches. Most contributions were recent, with only one paper published before 2002; this indicates that scientific attention on forest therapy and stress reduction is a very new topic. In terms of experimental settings, most studies involved on-site data collection, which means that the recruited sample was brought to the forest to allow forest immersion. A smaller share of experiments were conducted in a lab setting by means of virtual reality (VR) devices that simulated natural environments.

**Figure 2:**
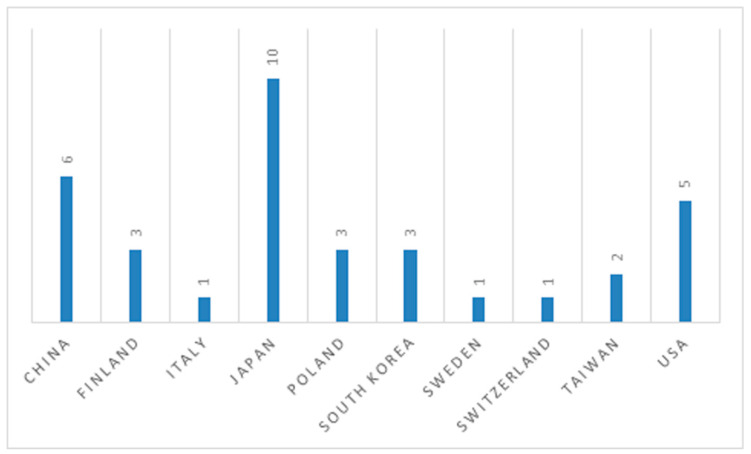
Geographical distribution of the studies.

Figure 2 shows the geographical distribution of the studies. Japan is the most represented country with 10 studies. China and the USA are represented by six and five studies, respectively, while South Korea, Finland and Poland each have three studies. Other countries where forest therapy studies were conducted are Italy, Sweden, Switzerland and Taiwan, all with a smaller number of one or two studies.

### 3.1. Quantitative Review (Text Analysis)

The stress index of MDS output (0.092) indicates a positive correlation between the input matrix and Sammon’s map [53].

**Figure 3:**
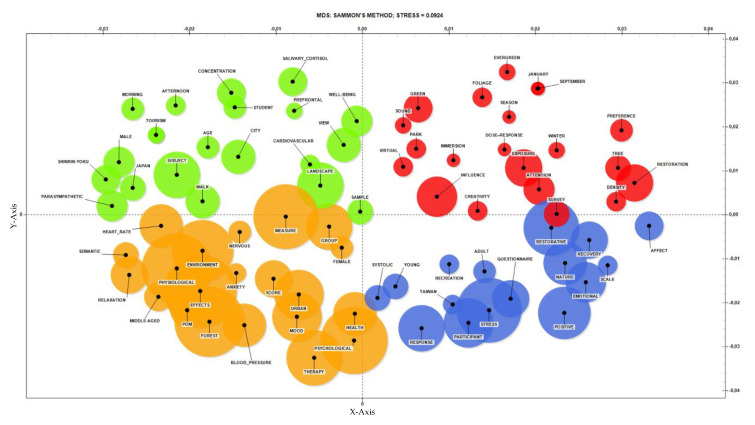
Sammon’s map showing relationships among lemmas (different colours denote the four quadrants).

The map displayed in Figure 3 shows that research interest has, to date, concentrated on some topics. In the first quadrant, the influence of forest variables on stress relief is shown. Lemmas such as “tree” or “density” are related to “restoration” effects or “preference” of people. The “attention” level of interviewees as to the “exposure” to forests, in particular through the so-called “dose–response” effect are investigated with particular care. Forest seasonality was very influential on stress recovery (due to lemmas “season”, “foliage”, “evergreen”, “winter” or specification of month of the year). Immersion of people in forests—on-site or using new “virtual” reality technologies—is indicated on the map. Some studies report the importance of the “sound” of forests for stress recovery (analyzed in literature, particularly in “park” and urban green areas). The second quadrant focuses on a cluster of lemmas related to physiological indicators of “well-being”. Some scientific papers use “cardiovascular” parameters, “salivary cortisol”, “prefrontal” performances (i.e., activity in highly stress-related area of brain) or “parasympathetic” nervous system activation to define the status of people in the case of forest exposition. Forest variables are included in the specific sense of “view” and “landscape” evaluation. Activities (“walk”) and performances (“concentration”) are investigated in general terms (“subject”) or for specific age and status (“student”). An interesting topic appears in this quadrant, that is, the “tourism” tendency of forest bathing (“Shinrin-yoku”) is emphasized, particularly for “Japan”. Undertaking analyses at different times of the day seems to be relevant for stress recovery (“morning” and “afternoon” lemmas). The third quadrant introduces the basic concept linked to “forest”, “therapy”, “health” and “anxiety” as well as the evaluation of the most investigated “physiological” parameters (“heart rate” and “nervous” trend). However, a focus on psychological analysis is here outlined by the terms “score” (typical of questionnaires), “mood” and “POMS” scale. The last quadrant also designates a cluster composed by general lemmas describing both psychological (“questionnaire”, “scale”) and physiological (“systolic”) terms, interviewed group (“young”, “adult”) and area (“Taiwan”). The preliminary text mining is based on a limited number of papers in respect to the existing application of similar methods in the literature [54]; however, it provides a good comparison with qualitative literature reviews described in this paper, facilitating replicability of the analysis for different countries or temporal trends. In future works, additional quantitative evaluation could be introduced.

### 3.2. Performance Measurement

The effectiveness of forests for relaxation and rest compared to urban areas or control groups has been tested with two families of indicators: (1) self-rated measures and (2) physiological outcomes.

Self-rated measures are subjective answers of participants to a set of questions that capture the perceived mood after stimuli administration. There are several ready-to-use questionnaires for this purpose. The most common scale in the sample was the Profile Of Mood States (POMS), whose purpose is to evaluate individual moods associated with certain forest exposure [37,38,39]. The Positive And Negative Affect Schedule (PANAS) is instead one of the oldest but not so popular scales available [55], and was used by Bielinis et al. [37,51] and Park et al. [36] for the evaluation of the therapeutic effects of forests. The evaluation of rest is conducted with a specific scale called the Restoration Outcome Scale (ROS), which is composed of six items [56]. In the forest therapy literature, the use of ROS is relatively popular [27,30,37,39,47]. Less popular measures are the Subjective Vitality Scale (SVS) [37], the Perceived Stress Scale (PSS) [37], the emotional well-being scale (RAND 36) [29], the semantic differential method (SDM) [32], total mood disturbance (TMD) [32], the Maslach Burnout Inventory (MBI) [31] and the restored ability to work (RAW) [57]. Interestingly, one contribution investigated, among other indicators, the “fluid procrastination”, which indicates a pessimistic attitude to complete a job. The psychological stress measure (PSM-9) is a popular approach to measure stress [58], which uses a set of nine indicators; however, none of the studies considered in this review implemented investigations based on PSM-9. This result is a potential indication that most research in forest therapy considered rest potential and mood induced by forests with specific scales such as POMS and ROS, rather than measures of stress levels. Lastly, some papers captured self-rated measures with scales that were not previously validated in the literature [22,40].

With respect to physiological measures, saliva samples for cortisol and amylase represent the most common indicator [18,24,26,28,30,31,38]. Two main other measures are blood pressure and heartbeat [17,21,25,34,37]. Often cortisol, blood pressure and heartbeat are measured simultaneously. Interestingly a minority of other contributions utilized indicators of natural killer cells [31]. Another important indicator of stress is the analysis of cerebral activity, collected with electroencephalogram devices [33,47,52].

### 3.3. Forests’ Effects of Relaxation and Rest

An initial interesting result is that all studies report a positive impact of exposure to forest environments on measures related to stress and rest, regardless of the indicator used. Several Japanese studies, where forest therapy is often referred to as “Shinrin-yoku” (taking in the atmosphere of the forest), indicate that spending time in forests helps in reducing cortisol levels and blood pressure, as well as contributing to a more stable heartbeat [16,23,24]. Several Chinese studies confirmed a positive association between stress relief and forest recreation [27,32,59]. Moving to Europe, in Nordic countries, numerous studies demonstrated that in experiments that compare stress levels between urban areas and forests, participants always show lower stress levels when exposed to forested areas [29,30]. Similar findings were obtained in Italy using a combination of virtual reality, EEG and ROS scales [47]. Another interesting application of virtual reality was offered by Annerstedt et al. [26], who compared three groups exposed to an urban setting, a forest and a forest including sounds of nature, respectively. Results indicated that forest relaxation is highest when coupled with sounds of nature. Beil and Hanes conducted a study in the USA where relaxation effects were also confirmed [18].

While no studies reported a failure of forest for relaxation, some studies reported a bad performance of some of the indicators, in particular those related physiological outcome. Two studies in particular reported a negligible or absent effect of forest exposure on lowering saliva cortisol levels [18,30]. Authors attributed the result to the short duration of the stimuli, arguing that cortisol response to external stimuli is slow, and that it requires long exposure of respondents to the treatments.

### 3.4. Influence of Forest Characteristics

Results indicated that, to date, most scientific attention has concentrated on stress levels associated with the urban–forest dichotomy, and that all contributions confirm good forest performance in relation to stress reduction. However, forests are not all the same and vary based on tree species composition, structure, canopy cover, dead wood and other variables that shape forest landscapes. Therefore, the potential of forests to relieve stress may be unevenly distributed across different forest stands, with some forests more appropriate than others. In this respect, the literature is scarcer of contributions, and only five studies collected in this review considered forest characteristics in their experimental design. A pioneer study in this regard was made by Ulrich et al. [17], who compared blood pressure and muscle tension of participants across six different forests. However, the study did not consider single forest characteristics and focused only on different types of forest landscape. Forest density was considered by Chiang et al. [33], whose results indicated that forest density correlates with stress levels. In their study, high density forests caused higher attention levels of participants, but medium density forests were reported to be favoured according to self-rated measures. Bielinis et al. investigated seasonal variations of forest stands for stress recovery, comparing the results of forest therapy between spring and winter [37]. Sacchelli et al. [47] implemented a virtual reality study where four different forest types were compared in winter, and they found that coniferous forests and Douglas fir in particular were more appropriate for stress relief purposes. In China, another virtual reality study by Wang and Zhao [52] confirmed that evergreen trees are more effective to maximize the stress relief potential of forests.

## 4. Knowledge Gaps

Despite a few number of papers currently focusing on the topic “forests for therapy”, from a methodological point of view, additional quantitative evaluation could be introduced, especially in the case of largely investigated techniques (e.g., scales applied in questionnaires, neuroscientific tools, visitors preferences, etc.). Text mining gives an objective picture of the analyzed theme, facilitating the summary and extraction of take-home messages. It allows replicability in different times and for a diversified study area. However, a specific guideline on how to use the potentially large amount of available grey literature about forests and health should also be defined.

A certain lack of knowledge is detected in regard to the social benefits—derived from forest activities—that influence mental and physical well-being of individuals, such as strengthening or developing new social relationships [60]. Specific attention should be paid to the dose–response effect, concentrating in particular on differences between application of psychological and physiological measurement of stress relief as well as on the influence of frequency and duration of activities.

A final remark is related to the potential negative impacts of forests on people’s health. That is, risks due to allergenic reactions, pests, insects, falling branches and trees or wildfires should be considered—in particular in rural–urban interfaces—to allow the investigation of trade-offs among ecosystem services and disservices.

## 5. Conclusions

The issue of forest characteristics is relevant because the simple comparison of urban versus forest environments provides very little information to forest management. The available scientific evidence gives little information on the fundamental managerial question “How should the forest be managed to increase forest therapy potential?” For this reason, more research on forest characteristics associated with relaxation is necessary to better inform forest-therapy-oriented management. This aspect concerns forest types and tree species composition as well as age structure, cover density, amount of deadwood and, more generally, all the variables that have an impact on forest landscape.

## Figures and Tables

**Table 1:** Acronym of self-rated indicators used in primary studies.

Nomenclature	
PANAS	Positive And Negative Affect Schedule
POMS	Profile Of Mood States
SVS	Subjective Vitality Scale
PSS	Perceived Stress Scale
RAND 36	Emotional well-being scale
SDM	Semantic differential method
TMD	Total mood disturbance
STAI	State-Trait Anxiety Inventory
ROS	Restoration Outcome Scale
MBI	Maslach Burnout Inventory

**Table 2:** Studies included in the review.

Authors	Year	Source	Self-Rated Measures	Physiological Measures
Ulrich et al.	1991	[17]	-	blood pressure, muscle tension
Hartig et al.	2003	[21]	-	blood pressure
Hansmann et al.	2007	[22]	non-validated measures	-
Park et al.	2007	[23]	-	cortisol, electroencephalography (EEG)
Park et al.	2008	[24]	-	cortisol, heart beat
Park et al.	2009		-	cortisol, heart beat
Li et al.	2012	[25]	POMS	blood pressure, heart beat, urine dopamine, and cardiovascular and metabolic parameters
Annerstedt et al.	2013	[26]	-	cortisol, cardiovascular data
Beil and Hanes	2013	[18]	PSS	cortisol
Tsunetsugu et al.	2013	[27]	POMS, ROS	blood pressure, heart beat, nervous activity
Jiang et al.	2014	[28]	-	cortisol, skin conductance
Korpela et al.	2014	[29]	RAND 36	
Tirvainen et al.	2014	[30]	ROS	cortisol
Jung et al.	2015	[31]	MBI	cortisol, heartbeat, natural killer cell
Ochiai et al.	2015	[32]	SDM, POMS, TMD	blood pressure
Chiang et al.	2017	[33]	POMS	EEG
Ohe et al.	2017	[34]	POMS	blood pressure, heart beat
Park et al.	2017	[35]	PANAS	heart rate, prefrontal cortex activity
Song et al.	2017	[36]	SDM	heartbeat, blood pressure
Bielinis	2018	[37]	PANAS, POMS, ROS, SVS	
Chen et al.	2018	[38]	POMS, STAI	salivary amylase, blood pressure, heart beat
Bielinis et al.	2019	[39]	ROS, POMS	blood pressure, heartbeat
Lee et al.	2019	[40]	non-validated measures	-
Song et al.	2019	[41]	POMS, STAI	nervous activity, heartbeat
Wang et al.	2019	[42]	POMS	
Chia-Pin and Hsieh	2020	[43]	POMS, TMD	
Huang et al.	2020	[44]	-	skin conductance
Kim et al.	2020	[45]	POMS	
Kuper	2020	[46]	-	heartbeat, blood pressure
Sacchelli et al.	2020	[47]	ROS	EEG
Janeczko et al.	2020	[48]	POMS, PANAS, ROS, SVS	heart rate, blood pressure
Zeng et al.	2020	[49]	SDM	peripheral oxygen saturation, heartbeat, blood pressure
Jiang	2020	[50]	-	EEG, skin conductance, heartbeat, blood pressure
Bielinis et al.	2020	[51]	fluid procrastination	-
Wang and Zao	2020	[52]	-	EEG
